# lncRNA HCG11 suppresses cell proliferation in hormone receptor-positive breast cancer via SRSF1/β-catenin

**DOI:** 10.18632/aging.204468

**Published:** 2023-01-04

**Authors:** Dan Xie, Saiyang Li, Xuehui Wang, Lin Fang

**Affiliations:** 1 Changzhou Traditional Chinese Medicine Hospital Affiliated to Nanjing University of Chinese Medicine, Changzhou 213000, Jiangsu, P.R. China; 2 The Third Affiliated Hospital of Soochow University, Changzhou 213000, Jiangsu, P.R. China; 3 Department of Breast and Thyroid Surgery, Shanghai Tenth People’s Hospital, Tongji University School of Medicine, Shanghai 200072, P.R. China

**Keywords:** HCG11, cell proliferation, hormone receptor-positive breast cancer, SRSF1, β-catenin

## Abstract

Hormone receptor positive (HR-positive) breast cancer (BC) is the most common subtype of breast cancer. Despite adjuvant endocrine therapy and chemotherapy-based treatment, the therapeutic response is often not satisfactory in HR-positive BC patients. Therefore, elucidating the mechanisms that regulate the progression of HR-positive BC is urgently required to identify new therapeutic targets. Previously, HLA Complex Group 11 (HCG11), located on the major histocompatibility complex (MHC) region, was found to be abnormally expressed in a variety of tumor cells. However, the role of HCG11 in HR-positive BC cells has not been explored to date. In the current study, we found that HCG11 is downregulated in HR-positive BC tissues and cell lines. Both *in vitro* and *in vivo*, HCG11 acts as a tumor suppressor in HR-positive BC cells. Furthermore, the mechanistic details unraveled that HCG11 recruits Serine/arginine-rich splicing factor 1 (SRSF1) to target β-catenin mRNA for promoting the translation of β-catenin. Our study emphasizes the potential of HCG11 as a novel intervention target for HR-positive BC treatment.

## INTRODUCTION

The latest global cancer data show that breast cancer (BC) has become the most common malignancy in women worldwide in terms of the number of incidence and cancer-related mortality [1]. The molecular typology of BC is the basis of the current diagnosis and its treatment. There are different subtypes of BC based on the expression of hormone receptors (HR), including estrogen receptor (ER) and progesterone receptor (PR), and human epidermal growth factor receptor 2 (HER2) in the tumor cells [2, 3]. ER-positive or luminal tumors account for approximately two-thirds of all BCs [4]. Patients with HR-positive BC have a better prognosis than patients with HER2-enriched or basal-like BC. However, despite adjuvant endocrine therapy and chemotherapy-based treatment, the therapeutic response is often not satisfactory in HR-positive BC patients [5]. Therefore, exploring the underlying mechanisms of the development and progression of HR-positive BC and identifying new therapeutic targets is of great clinical importance for efficient prognosis and survival of BC patients.

Long non-coding RNAs (lncRNAs) are non-coding RNA (ncRNAs) consisting of more than 200 nucleotides [6]. LncRNAs can be categorized as antisense, intergenic, intronic, or overlapping RNAs based on how closely they are associated with the protein-coding genes [7]. Various studies have shown that lncRNAs are involved in the development, metastasis, and recurrence of almost all malignant tumors. HLA Complex Group 11 (HCG11), located in the major histocompatibility complex (MHC) region, was found to be expressed abnormally in different types of tumors [8]. An in-depth study of HCG11 in different types of malignancies has revealed that the role of HCG11 appears to be inconsistent in cancers. For instance, HCG11 inhibits the growth of human osteosarcoma by increasing the expression of p27 Kip1 [9]. HCG11 expression is downregulated in lung adenocarcinoma tissues which inhibits tumor progression by modulating the IGF2BP2/LATS1 axis [10]. On the other hand, HCG11 may encourage the proliferation and migration of gastric cancer cells by targeting the miR-1276/CTNNB1 cascade and triggering the Wnt signaling pathway [11]. In addition, HCG11 promotes nasopharyngeal cancer development by regulating the miRNA-490-3p/MAP3K9 pathway [12]. Interestingly, HCG11 was differentially expressed in different BC subtypes and participated in distinct regulatory networks [13]. In a previous study, the expression of HCG11 was found to be associated with ER status, which might be useful in hormone replacement therapy [14]. Further, higher HCG11 expression was observed in basal-like type patients as compared to luminal A type patients. By targeting Sp1, HCG11 may regulate the invasion and survival of TNBC cells [15]. Though the differential expression of HCG11 in different BC subtypes has been documented, its precise role in HR-positive BC is not well understood to date.

In the current study, a decreased expression of HCG11 was observed in HR-positive BC cells. Further, by exploring the potential regulatory mechanisms of HCG11, it was found that HCG11 inhibits tumor growth by regulating the SRSF1/β-catenin cascade in HR-positive BC cells.

## RESULTS

### The expression of HCG11 was decreased in HR-positive BC cells

**Figure 1:**
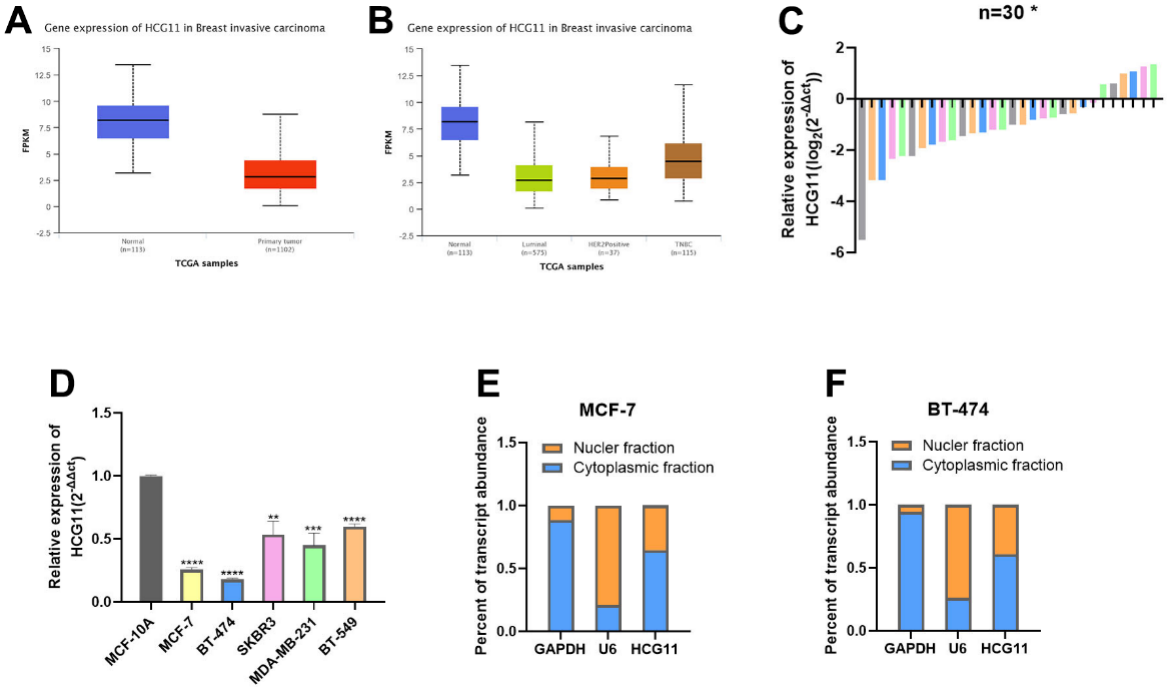
**The expression of HCG11 was decreased in HR-positive BC cells.** (**A**, **B**) Expression of HCG11 in BC tissues according to the data from UALCAN database. (**C**) Relative expression of HCG11 in HR-positive BC tissues. (**D**) Relative expression of HCG11 in HR-positive BC cell lines. (**E**, **F**) Expression levels of cytoplasmic control transcripts (GAPDH), the nuclear control transcript (U6), and HCG11 were determined by qRT-PCR in the cytoplasmic and nuclear fractions of MCF-7 and BT-474. *p<0.05, **p<0.01, ***p<0.001, **** p<0.0001.

Based on the cancer histology data obtained from the UALCAN database, we found that HCG11 was expressed at significantly low levels in BC tissues, and its expression was lower in HR-positive BC tissues than that in HER2-enriched and basal-like BC tissues (Figure 1A, 1B). Next, we examined the expression levels of HCG11 in 30 pairs of HR-positive BC tissues and adjacent normal tissues. Of these, the HCG11 expression was significantly lower in 24 HR-positive BC tissues than in the adjacent normal tissues. These findings were consistent with the UALCAN database (Figure 1C). To further investigate the correlation between HCG11 expression and clinicopathological variables in HR-positive BC patients, the relevant clinical information of 30 BC patients was recorded. We observed that low HCG11 expression was positively correlated with the tumor size, but not with age, TNM stage and lymph node metastasis in HR-positive BC patients (Table 1).

**Table 1:** The relationship between the expression of HCG11 and various clinicopathological variables in HR-positive BC patients.

**Patients characteristics**	**Total**	**HCG11 expression**		**P value**
	**(*n=30)***	**High (*n=6)***	**Low (*n=24)***	
**Age**				>0.9999
<60	13	3	10	
≥60	17	3	14	
**TNM stage**				0.5569
I and II	26	6	20	
III and IV	4	0	4	
**Tumor size(cm)**				0.0237*
≤2	17	6	11	
>2	13	0	13	
**Lymph node metastasis**				0.1405
negative	21	6	15	
positive	9	0	9	

* p < 0.05.

Furthermore, the expression levels of HCG11 were examined in different cell line subtypes, which revealed that HCG11 expression was lowest in the HR-positive BC cell lines MCF-7 and BT-474, and highest in the basal-like BC cell lines BT-549 (Figure 1D). The cytoplasmic nucleus separation assay indicated that HCG11 was expressed in both nucleus and cytoplasm in HR-positive BC cells, though the higher expression was observed in the cytoplasm (Figure 1E, 1F).

### HCG11 inhibited the proliferation of HR-positive BC cells *in vitro*

**Figure 2:**
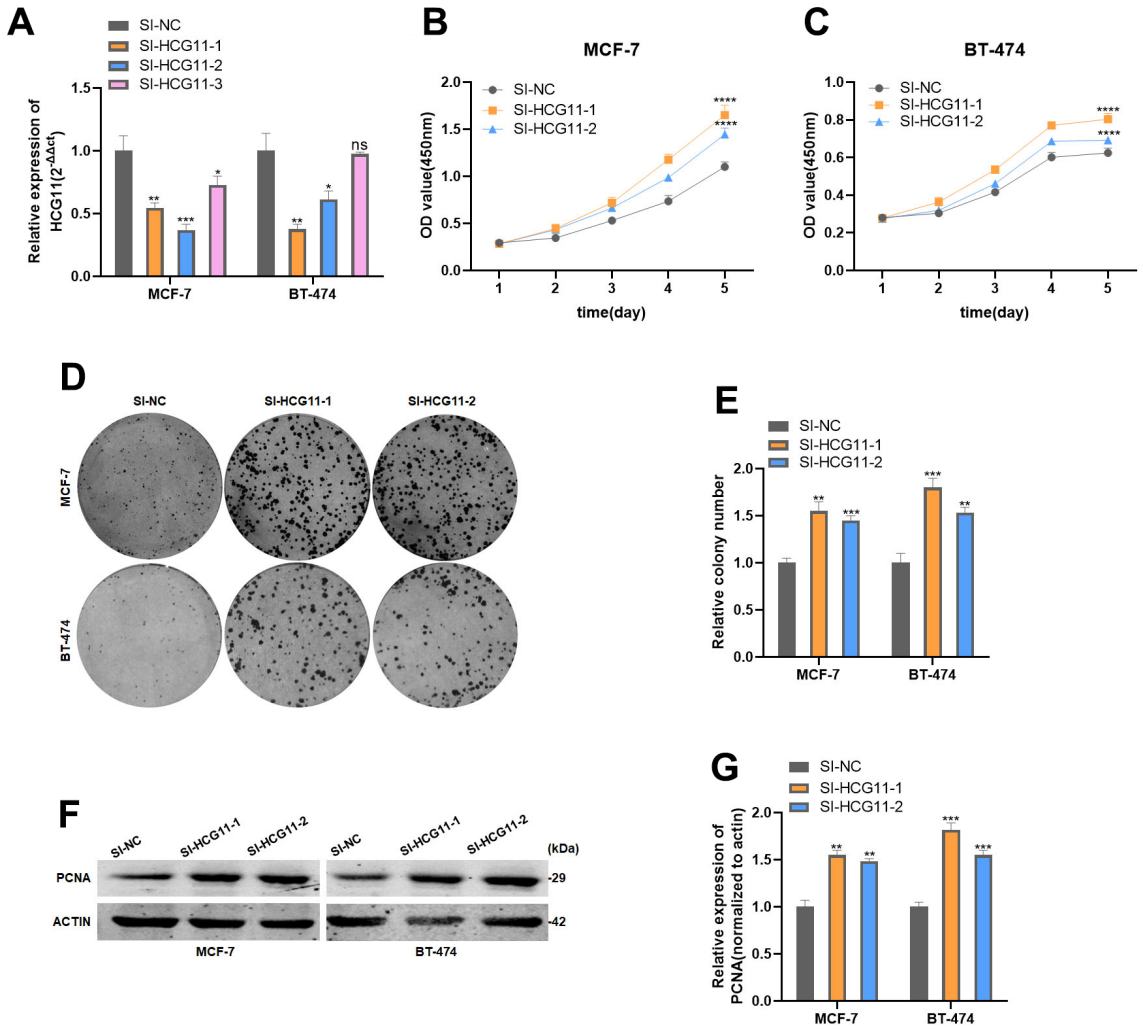
**HCG11 inhibited the proliferation of HR-positive BC cells *in vitro*.** (**A**) Expression of HCG11 was confirmed by qRT-PCR in MCF-7 and BT-474 cell line transfected with SI-NC or SI-HCG11. (**B**, **C**) Effect of SI-HCG11 on proliferation in MCF-7 and BT-474 cell line by CCK8 assay. (**D**, **E**) Effect of SI-HCG11 on proliferation in MCF-7 and BT-474 cell line by colony formation assay. (**F**, **G**) Effect of SI-HCG11 on proliferation in MCF-7 and BT-474 cell line by western blot. *p<0.05, **p<0.01, ***p<0.001, **** p<0.0001, ns represents p>0.05.

Considering the significantly low expression of HCG11, we sought to explore the precise role of HCG11 in HR-positive BC cells. We first designed three small interfering RNAs (siRNAs) targeting HCG11 (SI-HCG11-1, SI-HCG11-2, and SI-HCG11-3) to knock down its expression. Based on the interference efficiency depicted in Figure 2A, we selected SI-HCG11-1 and SI-HCG11-2 for subsequent studies. CCK8 assays showed that knockdown of HCG11 after si-HCG11 transfection promoted cell proliferation in MCF-7 and BT-474 cell lines (Figure 2B, 2C). The results of the colony formation assay further confirmed this observation (Figure 2D, 2E). Meanwhile, the protein expression of cell proliferation marker, PCNA, was up-regulated after the depletion of HCG11 (Figure 2F, 2G), further highlighting the role of HCG11 in inhibiting the proliferation of HR-positive BC cells *in vitro*.

### HCG11 could bind to SRSF1

**Figure 3:**
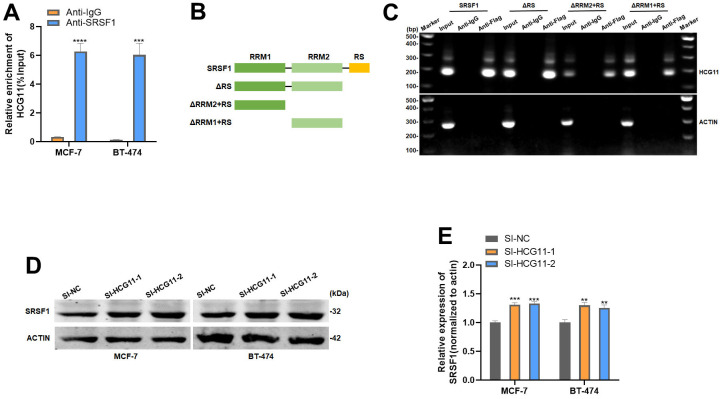
**HCG11 could bind to SRSF1.** (**A**) Anti-SRSF1 RIP assay was applied to validate the binding of SRSF1 to HCG11. (**B**) Full-length and truncated SRSF1 plasmid with FLAG-tagged vectors were constructed. (**C**) Anti-Flag RIP assay was applied to validate the binding of SRSF1 to HCG11. (**D**, **E**) Protein level of SRSF1 in MCF-7 and BT-474 cell line transfected with SI-HCG11. **p<0.01, ***p<0.001, **** p<0.0001.

We further investigated the potential molecular mechanisms by which HCG11 inhibits cell proliferation in HR-positive BC. The preliminary analysis using the bioinformatics starbase database predicted the interaction between HCG11 and Serine/arginine-rich splicing factor 1 (SRSF1). To validate this possibility, we first performed the RIP assay, which revealed that HCG11 was significantly enriched in the mixtures immunoprecipitated with the anti-SRSF1 antibody (Figure 3A). SRSF1 is an RNA-binding protein consisting of one RS domain and two RNA recognition motifs. Of these, the latter usually plays a major role in RNA binding. We created various mutants of the SRSF1 domain to pinpoint the location where SRSF1 binds to HCG11. (Figure 3B). After infecting the 293T cells with the above-mentioned mutants, RIP and subsequent PCR assays were performed. The results indicated that HCG11 bound to the RRM1 and RRM2 domain of SRSF1 (Figure 3C). Subsequently, we observed that depletion of HCG11 increased the protein level of SRSF1, elucidating that HCG11 is directly responsible for the upregulation of SRSF1 protein (Figure 3D, 3E).

### SRSF1 expression was increased in HR-positive BC

**Figure 4:**
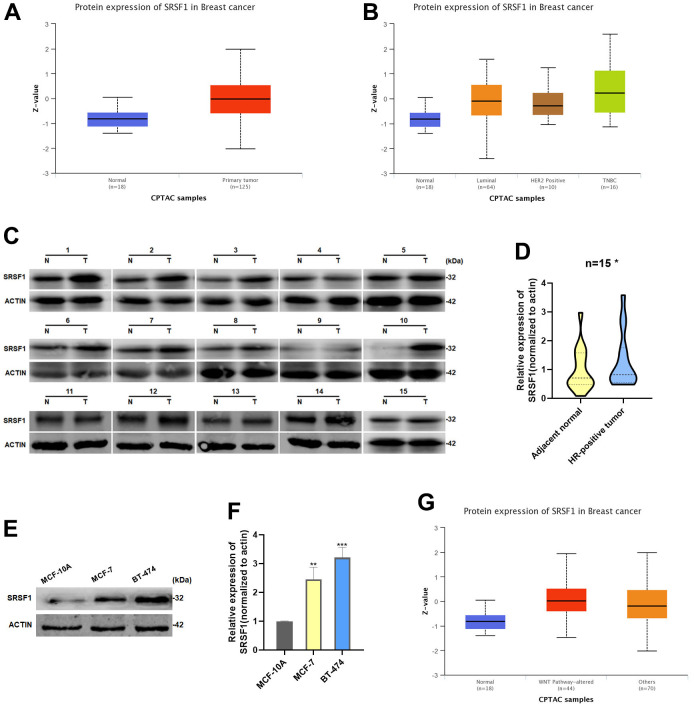
**SRSF1 expression was increased in HR-positive BC.** (**A**, **B**) Expression of SRSF1 in BC tissues according to the data from UALCAN database. (**C**, **D**) Relative expression of SRSF1 in HR-positive BC tissues. (**E**, **F**) Relative expression of SRSF1 in HR-positive BC cell lines. (**G**) Expression of SRSF1 in WNT-Pathway altered BC tissues according to the data from UALCAN database. *p<0.05, **p<0.01, ***p<0.001.

As demonstrated in the UALCAN database, SRSF1 protein was expressed at a significantly high level in HR-positive BC tissues (Figure 4A, 4B). We extracted total protein from 15 pairs of HR-positive BC tissues and adjacent normal tissues, and detected the expression of SRSF1. In consistence with the data obtained from the UALCAN database, we discovered that the expression of SRSF1 was significantly high in HR-positive BC tissues (Figure 4C, 4D). In addition, the SRSF1 protein also showed high expression in HR-positive BC cell lines (Figure 4E, 4F). Interestingly, we found that the high levels of SRSF1 in BC tissues with altered Wnt pathway (Figure 4G), suggesting that SRSF1 might play a crucial role in regulating the Wnt/catenin signaling pathway.

### SRSF1 promoted Wnt signaling via upregulating β-catenin expression

**Figure 5:**
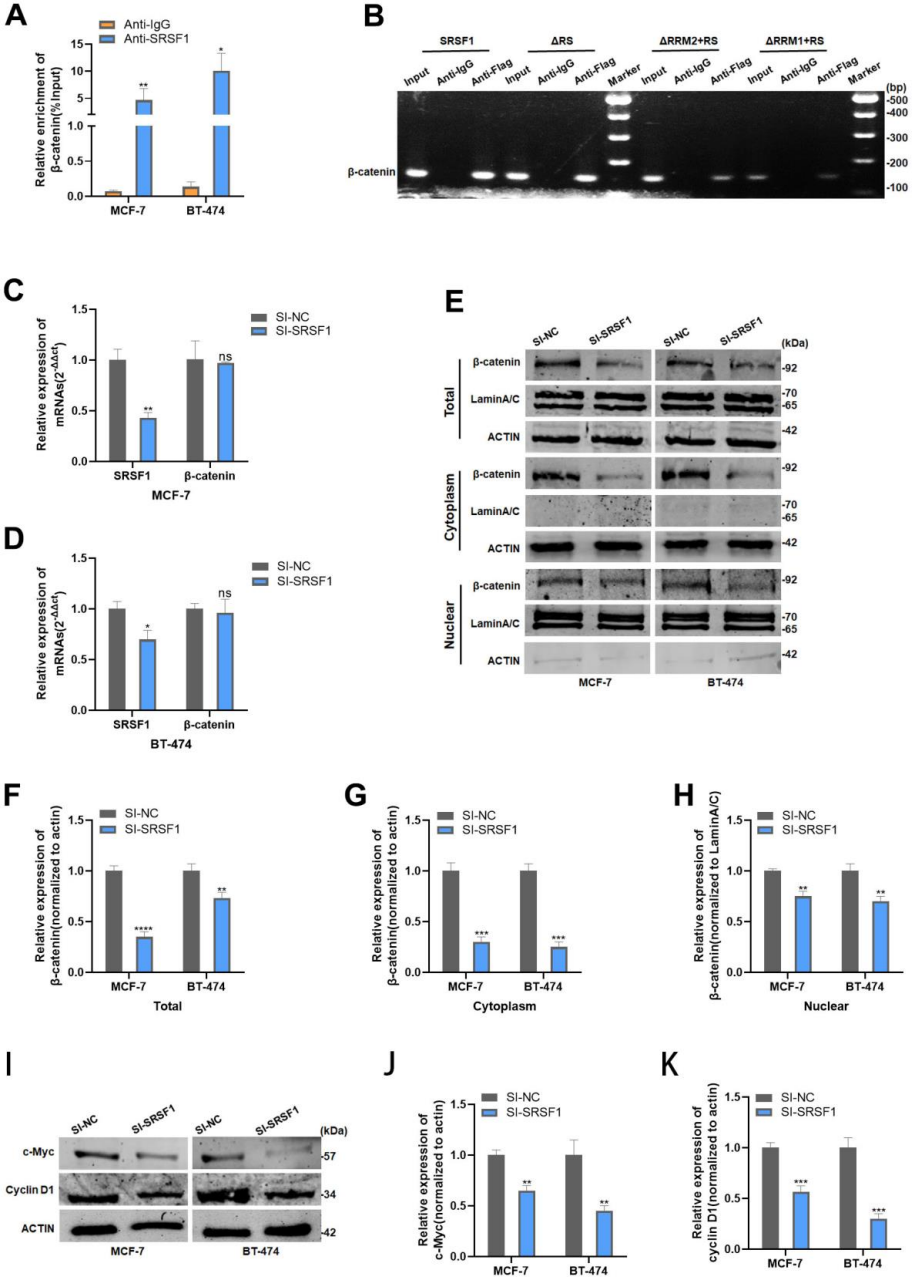
**SRSF1 promoted Wnt signaling via upregulating β-catenin expression.** (**A**) Anti-SRSF1 RIP assay was applied to validate the binding of SRSF1 to β-catenin mRNA. (**B**) Anti-Flag RIP assay was applied to validate the binding of SRSF1 to β-catenin mRNA. (**C**, **D**) mRNA level of β-catenin in MCF-7 and BT-474 cell line transfected with SI-SRSF1. (**E**–**H**) Protein level of β-catenin in MCF-7 and BT-474 cell line transfected with SI-SRSF1. (**I**–**K**) Protein level of c-Myc and cyclin D1 in MCF-7 and BT-474 cell line transfected with SI-SRSF1. *p<0.05, **p<0.01, ***p<0.001, **** p<0.0001, ns represents p>0.05.

RIP analysis revealed that β-catenin mRNA was significantly enriched in the mixture immunoprecipitated with anti-SRSF1 antibody, indicating the ability of SRSF1 to interact with β-catenin mRNA (Figure 5A). Further, RIP and subsequent PCR assay elucidated that β-catenin mRNA interacts with both RRM1 and RRM2 domains of SRSF1 protein (Figure 5B). Subsequently, we discovered that SRSF1 only affects the protein levels of β-catenin, not the mRNA levels (Figure 5C, 5D). We further explored the effect of disrupting SRSF1 expression on the subcellular distribution of the β-catenin protein. Knockdown of SRSF1 expression resulted in a decrease in the protein levels of β-catenin in both the nucleus and cytoplasm in HR-positive BC cells (Figure 5E–5H). In addition, the target proteins of β-catenin, cell cycle protein D1 and c-Myc, were also inhibited in SRSF1-depleted BC cells (Figure 5I–5K).

### HCG11 suppressed β-catenin expression by binding to SRSF1

**Figure 6:**
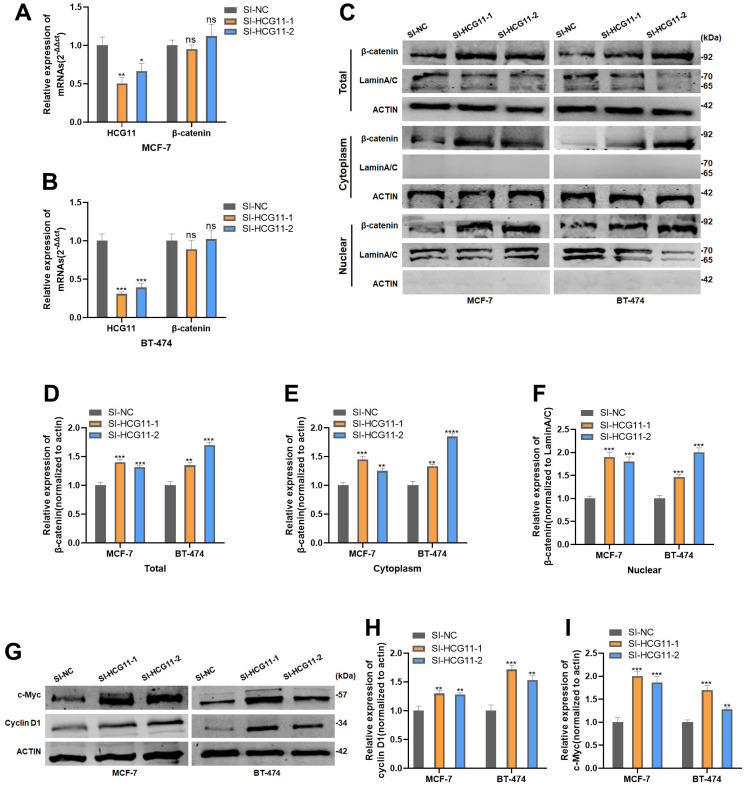
**HCG11 suppressed β-catenin expression by binding to SRSF1.** (**A**, **B**) mRNA level of β-catenin in MCF-7 and BT-474 cell line transfected with SI-HCG11. (**C**–**F**) Protein level of β-catenin in MCF-7 and BT-474 cell line transfected with SI-HCG11. (**G**–**I**) Protein level of c-Myc and cyclin D1 in MCF-7 and BT-474 cell line transfected with SI-HCG11. *p<0.05, **p<0.01, ***p<0.001, **** p<0.0001, ns represents p>0.05.

Since SRSF1 showed the binding ability to both HCG11 and β-catenin mRNA, we reasonably proposed the following hypothesis: HCG11 might compete with β-catenin mRNA for binding to SRSF1, thereby affecting the translation of β-catenin mRNA. To verify our hypothesis, both mRNA and protein levels of β-catenin were detected in HR-positive BC cells transfected with SI-HCG11. The findings demonstrated that despite the interference of HCG11, the mRNA level of β-catenin was unaffected (Figure 6A, 6B), but its total protein level, cytoplasmic fraction, and nuclear fraction were dramatically increased (Figure 6C–6F). Moreover, the protein levels of cyclin D1 and c-Myc were also altered in consistence with the β-catenin level after HCG11 interference (Figure 6G–6I).

### Overexpression of SRSF1 eliminated the antitumor effect of HCG11 on HR-positive BC cells

**Figure 7:**
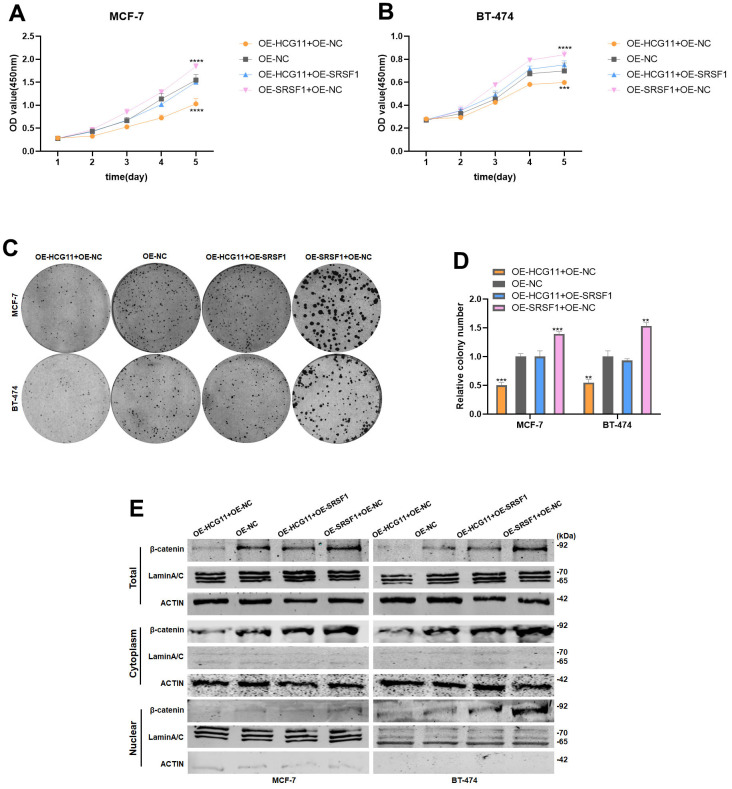
**Overexpression of SRSF1 eliminated the antitumor effect of HCG11 on HR-positive BC cells.** (**A**, **B**) SRSF1 rescued the suppressive effects of HCG11 on MCF-7 and BT-474 cell proliferation by CCK8 assay. (**C**, **D**) SRSF1 rescued the suppressive effects of HCG11 on MCF-7 and BT-474 cell line proliferation by colony formation assay. (**E**) SRSF1 rescued the low expression of β-catenin protein level caused by HCG11 by Western blot. **p<0.01, ***p<0.001, **** p<0.0001.

To further demonstrate that HCG11 modulates the protein level of β-catenin by binding to SRSF1, we co-transfected HCG11 plasmid and SRSF1 plasmid into HR-positive BC cells. Next, the cell proliferation experiments demonstrated that the overexpression of SRSF1 could effectively restore the cell proliferation capacity which was reduced due to the overexpression of HCG11 (Figure 7A–7D). More importantly, overexpression of SRSF1 could partially rescue the reduction of β-catenin protein level caused by the overexpression of HCG11, further demonstrating that HCG11 inhibited β-catenin expression via binding to SRSF1 (Figure 7E).

### HCG11 inhibited the proliferation of HR-positive BC cells *in vivo*

**Figure 8:**
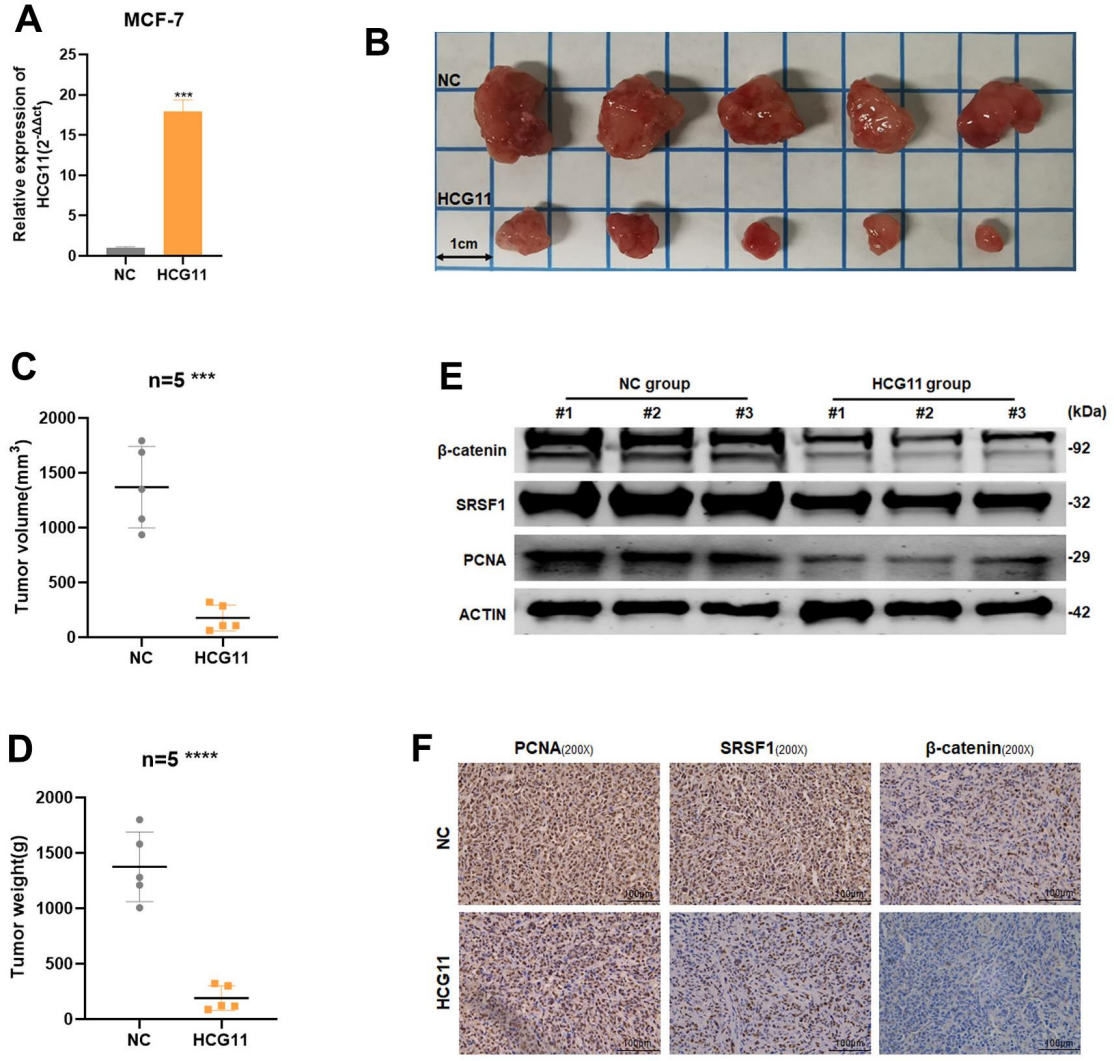
**HCG11 inhibited the proliferation of HR-positive BC cells *in vivo*.** (**A**) The transfection efficiency in MCF-7 cells were examined by qRT-PCR. (**B**) Representative images of xenograft tumors in nude mice. (**C**) Average tumor size of nude mice. (**D**) Average tumor weight of nude mice. (**E**) Extract protein from tumors and measuring the expression of PCNA, SRSF1 and β-catenin by Western blot. (**F**) Immunohistochemistry (IHC) staining of PCNA, SRSF1 and β-catenin in xenografts tumors. ***p<0.001, **** p<0.0001.

Using a lentiviral vector, we developed MCF7 cells lines stably expressing HCG11 to explore the biological role of HCG11 *in vivo* (Figure 8A). A xenograft mouse model was used to examine how HCG11 affects tumor growth. The findings unraveled that, in comparison to the negative control, overexpression of HCG11 could dramatically reduce the tumor size and weight (Figure 8B–8D). Subsequently, using western blot and IHC analyses, the expression of PCNA, SRSF1, and β-catenin was detected. The HCG11 group showed lower levels of PCNA, SRSF1, and β-catenin expression (Figure 8E, 8F). Taken together, we conclude that HCG11 inhibited the progression of HR-positive BC both *in vitro* and *in vivo*.

## DISCUSSION

The Major Histocompatibility Complex (MHC) is encoded by the HLA (Human Leukocyte Antigen) gene complex [16]. In previous studies, HLA genes were found to be associated with organ transplantation, immunotherapy, and tumorigenesis [17, 18]. The HLA Complex Group (HCG) is one of the numerous lncRNAs found in the MHC region in addition to the coding RNA. HCG18 is involved in the regulation of various physiological and pathological changes in papillary thyroid cancer, including cell proliferation, invasion, apoptosis, and epithelial-mesenchymal transition [19]. HCG22 prevents the proliferation and metastasis of bladder cancer cells by controlling PTBP1 [20]. It has been documented previously that HCG11 might help in the invasion and survival of TNBC cells by acting on Sp1 [15]. Though the differential HCG11 expression has been demonstrated in different BC subtypes, its precise role in the proliferation of HR-positive BC is not well understood.

In the current study, low expression of HCG11 was observed in consistence with the data obtained from the UALCAN database. In addition, in HR-positive BC patients, decreased expression of HCG11 was positively correlated with tumor size. The bioinformatics database predictions suggested that SRSF1 is a potential interacting protein for HCG11. Further, we confirmed using RIP analysis that HCG11 could reduce the protein levels of SRSF1 in HR-positive BC cells by directly binding to SRSF1.

The SR proteins (serine/arginine splicing factor, SRSFs) are important RNA-binding proteins that regulate RNA splicing, transport, and translation [21]. Although SR proteins are a class of proteins that are primarily located in the nucleus, but a recent study found that SR proteins could translocate from the nucleus to the cytoplasm [22]. Proto-oncogene SRSF1, formerly known as SF2/ASF, has been found to be overexpressed in various malignancies [23, 24]. Several mechanisms have been proposed to explain the possibility of how SRSF1 regulates tumorigenesis and progression. For example, the splicing factor SRSF1 was shown to accelerate the progression of BC via oncogenic splice switching of PTPMT1 [25]. SRSF1 regulates the SRA1 pre-mRNA splicing, which affected the invasion and metastasis of HCC cells [26]. These observations suggested that overexpression of SR proteins promotes the development of cancer through a variety of processes.

Wnt/β-catenin is a key signaling pathway involved in various physiological and pathological processes such as embryonic development, tissue homeostasis and tumorigenesis [27, 28]. While studying ER-positive BC patients who developed resistance to endocrine therapy, aberrant activation of Wnt/β-catenin signaling was observed [29]. The Wnt/β-catenin pathway involves many signaling responses, where the key event of signaling activation is the aberrant accumulation of β-catenin [30]. β-catenin is synthesized in large quantity in the cytoplasm and transferred to the nucleus, where it binds to the transcription factor TCF/LEF and promote the transcriptional activation of several downstream target genes with pro-cancer effects. Among the set of target genes, c-Myc and cyclin D1 were found have important roles and are most frequently mentioned in the previous reports [31]. According to the UALCAN database, SRSF1 is expressed at a high level in BC tissues with Wnt-pathway altered. In addition, SRSF1 can promote the synthesis and accumulation of β-catenin protein by recruiting and binding to its mRNA [32]. In the current study, we verified that SRSF1 could interact with β-catenin mRNA and promote the translation and accumulation of β-catenin protein in HR-positive BC cells. Based on the above findings, we reasonably conjectured that HCG11 could modulate the protein expression of β-catenin through SRSF1. Subsequent studies validated our proposed hypothesis that HCG11 can inhibit β-catenin accumulation in HR-positive BC cells. Moreover, SRSF1 overexpression can rescue the reduction in β-catenin protein caused by the overexpression of HCG11, supporting the idea that HCG11 works as an important cell proliferation inhibitor in HR-positive BC cells through the regulation of SRSF1/β-catenin.

Altogether, the current study confirmed the tumor-suppressive properties of HCG11 and illustrated its function in HR-positive BC cells. Meanwhile, our findings further demonstrate the promising role of HCG11 as a novel therapeutic target for HR-positive BC treatment.

## MATERIALS AND METHODS

### Cell culture and transfection

HR-positive BC cell lines MCF7 and BT474, human normal mammary epithelial cell line MCF10A and human embryonic kidney HEK293T were cultured in DMEM medium (Gibco, USA) or RPMI-1640 Medium (Gibco, USA). The siRNAs used in this study were designed and synthesized by Generay (China). The plasmids used in this research were designed and synthesized by IBSBio (China). Cell transfection was carried out using the Lipo8000™ Transfection Reagent (Beyotime, China).

### RNA extraction, qRT-PCR, PCR and agarose gel electrophoresis assay

The methods for RNA extraction, qRT-PCR, PCR, and agarose gel electrophoresis assays were performed by following the previous study [33]. Primers used in this study are as follows: HCG11-forward, 5’-GACTATTCACTGCGTGGTGG-3’ and HCG11-reverse, 5’-ATACTTTGAGCATTAAGACTTGT-3’; SRSF1-forward, 5’-CAACGATTGCCGCATCTACG-3’ and SRSF1-reverse, 5’-TCGAACTCAACGAAGGCGAA-3’; β-catenin-forward, 5’- CTGAGGAGCAGCTTCAGTCC-3’ and β-catenin-reverse, 5’-TCAAATCAGCTTGAGTAGCCA-3’; GAPDH-forward, 5’-ACCACAGTCCATGCCATCAC-3’ and GAPDH-reverse, 5’-TCCACCACCCGTTTGCTGTA-3’; β-Actin-forward, 5’-CTCCATCCTGGCCTCGCTGT-3’ and β-Actin-reverse, 5’-GCTGCTACCTTCACCGTTCC-3’.

### RNA binding protein immunoprecipitation (RIP) assay

The magnetic beads and anti-SRSF1 antibody (sc-33652, Santa Cruz Biotechnology, China) or anti-IgG antibody (AC005, Abclonal, China) were first co-incubated, followed by co-incubating the cell lysates with the prepared antibody-magnetic beads. Finally, the co-precipitated RNA was eluted and then analyzed by qRT-PCR and PCR assays. RIP assay was performed using the BersinBio™ RNA Immunoprecipitation Kit (BersinBio, China).

### *In vitro* cell proliferation assays

*In vitro* biological function assays were conducted as described previously [34].

### Protein extraction and Western blot analysis

Total protein was extracted using RIPA lysis buffer from Yeasen (China). Cytoplasmic and nuclear protein extraction was carried out using the Nuclear and Cytoplasmic Protein Extraction Kit (Yeasen, China). ACTIN and LaminA/C were used as internal controls for cytoplasmic and nuclear proteins, respectively. Primary antibodies used in this study were, c-Myc(ab32072, Abcam, USA), PCNA (A0264, ABclonal, China), SRSF1 (sc-33652, Santa Cruz Biotechnology, China), β-catenin (51067-2-AP, Proteintech, USA), cyclin D1(ab134175, Abcam, USA), LaminA/C (A0249, ABclonal, China), and ACTIN (AC026, ABclonal, China).

### Xenograft tumor assays

From the laboratory animal center of Shanghai, 10 Balb/c nude mice (5 weeks; 18-22g) were procured. Approximately, 1 × 10^6^ MCF-7 cells with stable HCG11 overexpression or negative control were injected into the second mammary fat of the mice (n = 5, each group). After the construction of an animal model, mice were executed by the cervical dislocation method. The mice tumors were photographed, measured, and weighted. The following formula was used to determine the size of the tumor:Volume (mm^3^) = 0.5 * width2 * length. The protocols involving animal models conformed with Tongji University’s ethics committee regulations.

### Immunohistochemistry (IHC)

Tumors were fixed in 4% paraformaldehyde immediately after removal from nude mice. After tissue embedding, slicing, dewaxing, and incubating with antibodies, images were taken at the proper magnification using a microscope (Leica Microsystems, Mannheim, Germany). Antibodies used in this experiment were, anti-PCNA (10205-2-AP, Proteintech, USA), anti-SRSF1(sc-33652, Santa Cruz Biotechnology, China) and anti-β-catenin (51067-2-AP, Proteintech, USA).

### Statistical analysis

The statistical analyses were performed using GraphPad Prism (version 8.0) statistical software. Data from at least three independent experiments were presented as mean ± SD. Student’s t-test was used to compare the variations between two groups. Two-way ANOVA was performed to analyze the results of the CCK8 assay. Fisher’s exact test was used to perform a correlation analysis between HCG11 expression and clinicopathological variables in BC patients. P-values less than 0.05 were considered statistically significant.

### Availability of data and material

UALCAN database was applied for RNA or protein expression analysis in this study. Starbase (https://starbase.sysu.edu.cn/index.php) was applied for RNA binding protein analysis of HCG11 in this study. For all other materials request, please contact the corresponding author at grace_579@163.com.
